# Depressive disorders after mild craniocerbral injuries in professional athletes

**DOI:** 10.1192/j.eurpsy.2025.1377

**Published:** 2025-08-26

**Authors:** ?. Syrmos

**Affiliations:** 1 Aristotle University, Thessaloniki, Greece

## Abstract

**Introduction:**

Craniocerebral injuries are serious traumatic situations with negative effects in the overall health and also in sports performace and health

**Objectives:**

Aim of this study is to present cases of deppresive disorders after mild craniocerebral injuries in professional athletes

**Methods:**

6 cases are presented. Range of age between 20 and 30 years old. All of them reported depressive disorders during the post traumatic period after craniocerebral injuries mainly during professional athletic activities

**Results:**

All of them they receive appropriate neurological, psychiatric, psycological and rehabilitation support and treatment. They managed to have a good outcome after 24 months follow up.

**Conclusions:**

The development of depressive disorders after such traumatic events remains a strong predictor of a variety of difunctions (social, personal, work etc). The emergence of depressive disorders in many cases remains unexplored and poorly understood. The effect into the the overall health remains a very important factor to investigate. The combination and collaboration of the various medical disciplines is essential in order to help young people and mainly professional athletes.

**Disclosure of Interest:**

None Declared

